# Catalytic Dyad Residues His41 and Cys145 Impact the Catalytic Activity and Overall Conformational Fold of the Main SARS-CoV-2 Protease 3-Chymotrypsin-Like Protease

**DOI:** 10.3389/fchem.2021.692168

**Published:** 2021-06-24

**Authors:** Juliana C. Ferreira, Samar Fadl, Adrian J. Villanueva, Wael M. Rabeh

**Affiliations:** Science Division, New York University Abu Dhabi, Abu Dhabi, United Arab Emirates

**Keywords:** COVID-19, SARS-CoV-2, 3-chymotrypsin-like protease, 3CLpro, catalytic dyad, thermodynamic stability, catalytic rate

## Abstract

Coronaviruses are responsible for multiple pandemics and millions of deaths globally, including the current pandemic of coronavirus disease 2019 (COVID-19). Development of antivirals against coronaviruses, including the severe acute respiratory syndrome-coronavirus 2 (SARS-CoV-2) responsible for COVID-19, is essential for containing the current and future coronavirus outbreaks. SARS-CoV-2 proteases represent important targets for the development of antivirals because of their role in the processing of viral polyproteins. 3-Chymotrypsin-like protease (3CLpro) is one such protease. The cleavage of SARS-CoV-2 polyproteins by 3CLpro is facilitated by a Cys145–His41 catalytic dyad. We here characterized the catalytic roles of the cysteine–histidine pair for improved understanding of the 3CLpro reaction mechanism, to inform the development of more effective antivirals against Sars-CoV-2. The catalytic dyad residues were substituted by site-directed mutagenesis. All substitutions tested (H41A, H41D, H41E, C145A, and C145S) resulted in a complete inactivation of 3CLpro, even when amino acids with a similar catalytic function to that of the original residues were used. The integrity of the structural fold of enzyme variants was investigated by circular dichroism spectroscopy to test if the catalytic inactivation of 3CLpro was caused by gross changes in the enzyme secondary structure. C145A, but not the other substitutions, shifted the oligomeric state of the enzyme from dimeric to a higher oligomeric state. Finally, the thermodynamic stability of 3CLpro H41A, H41D, and C145S variants was reduced relative the wild-type enzyme, with a similar stability of the H41E and C145A variants. Collectively, the above observations confirm the roles of His41 and Cys145 in the catalytic activity and the overall conformational fold of 3CLpro SARS-CoV-2. We conclude that the cysteine–histidine pair should be targeted for inhibition of 3CLpro and development of antiviral against COVID-19 and coronaviruses.

## Introduction

In the 21st century, coronaviruses have been the cause of three recent epidemics. Since its emergence in Wuhan, China, in December 2019, severe acute respiratory syndrome coronavirus 2 (SARS-CoV-2) has spread globally. It is responsible for millions of deaths worldwide, causing disease with influenza-like symptoms, including fever, shortness of breath, dry cough, fatigue, and diarrhea (Ciotti et al., 2019; Wu et al., 2020). The novel human coronavirus disease 2019 (COVID-19) caused by SARS-CoV-2 is one of the most challenging pandemics of the modern era. Even though several effective anti-SARS-CoV-2 vaccines have been developed, widespread vaccinations will take time because of logistical issues and additional unexpected resistance from some subpopulations that refuse to be vaccinated (Liu et al., 2020; Mulligan et al., 2020; Rut et al., 2020; Rawson et al., 2021). The efficacy (in terms of percentage) of the developed vaccines cannot be ruled out. Therefore, the need for antiviral drugs continues to be pressing for populations that do not have access to vaccines (or refuse their administration), lack antibodies against SARS-Cov-2, or are medically unfit to receive a vaccine.

The single-stranded RNA genome of SARS-CoV-2 consists of 14 open reading frames (ORFs) coding for 27 structural and nonstructural proteins (nsp) (Wu et al., 2020). Following entry and uncoating of the SARS-CoV-2, the host cell translation machinery expresses the two largest ORFs (ORF1a/b) into two overlapping polyproteins, pp1a and pp1ab (Rawson et al., 2021; V'Kovski et al., 2021). ORF1a encodes two viral cysteine proteases that are responsible for its proteolysis at 14 cleavage sites, to release 16 nsp (Rathnayake et al., 2020). The nsp proteins form the viral replication complex important for the replication of the viral genome and generation of new viruses (Anand et al., 2003; Kumar et al., 2020). The two proteases, papain-like protease (nsp3) and the main protease, 3-chymotrypsin-like protease (3CLpro; nsp5), are highly conserved among coronaviruses, including severe acute respiratory syndrome coronavirus (SARS-CoV) and Middle East respiratory syndrome coronavirus (MERS-CoV) that have emerged in 2002 and 2012, respectively. These enzymes represent attractive drug targets for the development of antivirals against COVID-19 as well as other coronavirus diseases (Helmy et al., 2020; Wu et al., 2020).

**FIGURE 1:**
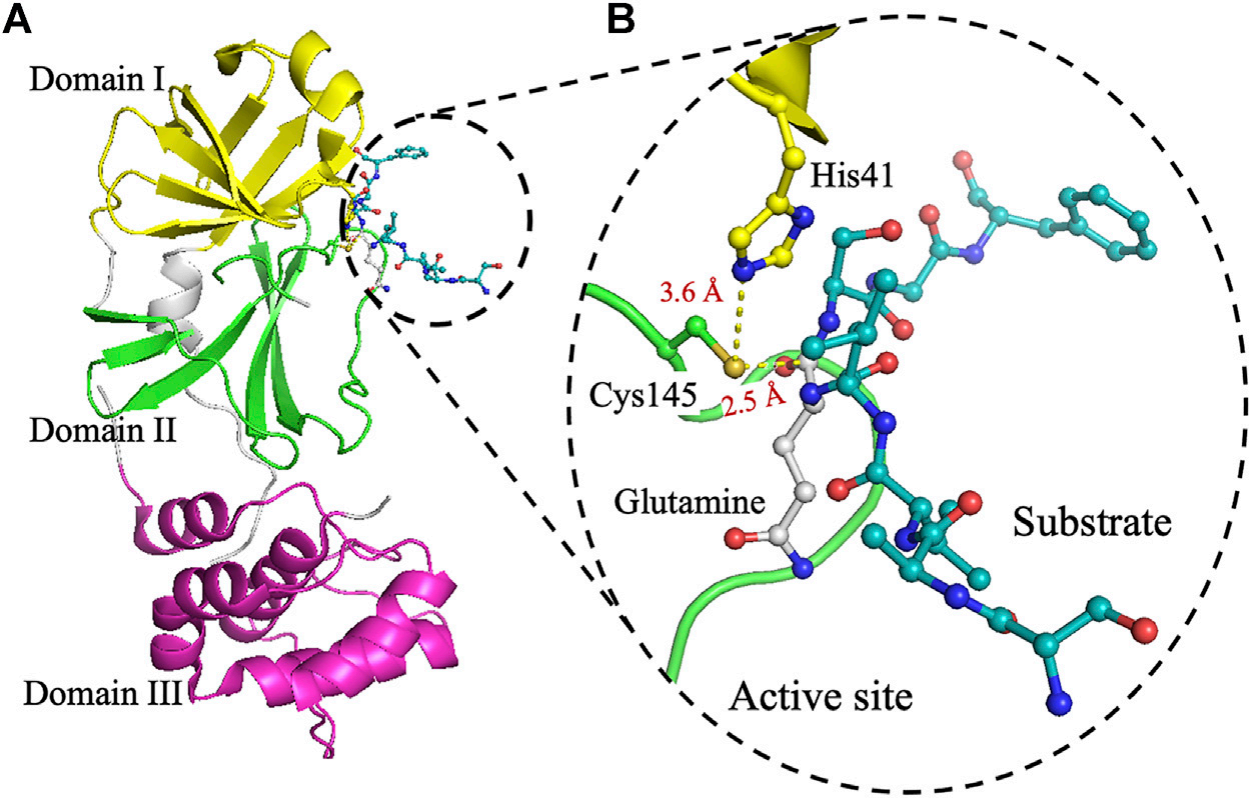
The crystal structure of 3CLpro. **(A)** Cartoon representation of 3CLpro of SARS-CoV-2 (PDB code 6Y2E) with domain I (residues 10–96) is shown in yellow, domain II (residues 102–180) in green, and domain III (residues 200–303) in pink. The peptide-substrate (blue) is shown in ball and stick representation, and it is located at the interface between domains I and II. **(B)** The active site of 3CLpro showing the peptide-substrate in blue and glutamine at P1-site is white. The catalytic residue Cys145, which is part of domain II, is 2.5 Å from the backbone carbonyl carbon of glutamine of the peptide-substrate. Residues of the catalytic dyad, His41 in domain I, is 3.6 Å from Cys145 in domain II. The figure was prepared using PyMol (Schrodinger LLC).

Homodimer is the active state of 3CLpro. It cleaves the viral polyprotein at 11 sites, at the conserved sequence Leu-Gln↓(Ser, Ala, Gly) (↓ marks the cleavage site) (Anand et al., 2003; Ratia et al., 2006; Goyal and Goyal, 2020; Jin et al., 2020; Zhang et al., 2020). Each monomer contains three domains (Figure 1A). The active site is located in a cleft between domain I (residues 8–99) and domain II (residues 100–183) (Anand et al., 2003; Muramatsu et al., 2016; Jin et al., 2020; Lee et al., 2020; Su et al., 2020; Zhang et al., 2020; Mody et al., 2021). Domains I and II adopt a fold reminiscent of a chymotrypsin-like fold, with six-stranded antiparallel β-barrels and a long loop (residues 184–199) connecting domain II to domain III (residues 200–300). Domain III contains five α-helices and assists in the dimerization of 3CLpro, mainly through a salt bridge interaction between Glu290 of one protomer and Arg4 of the other, as has been reported for 3CLpro from SARS-CoV (Anand et al., 2003; Wei et al., 2006; Wu et al., 2013). The dimeric structure is required for 3CLpro activity because interactions at the dimer interface near the active site stabilize the substrate in the appropriate binding conformation (Hsu et al., 2005; Barrila et al., 2006; Chen et al., 2008; Barrila et al., 2010). In addition, a short 3_10_-helix formed by a Ser139–Leu141 loop is twisted in the monomeric form to block access to the active site (Li et al., 2016).

3CLpro catalyzes the cleavage of the protein substrate using the catalytic dyad His41 and Cys145 (Huang et al., 2004; Ratia et al., 2006; Zhang et al., 2020). For better analysis of the interactions in the active site, the peptide substrate (SAVLQSGF) of 3CLpro from Porcine epidemic diarrhea virus (PEDV), PDB code 4ZUH, was docked into the structure of 3CLpro from SARS-CoV-2, PDB code 6Y2E (Figure 1B) (Ye et al., 2016; Zhang et al., 2020). Upon substrate binding, His41 deprotonates the thiol side chain of Cys145 to promote its nucleophilic attack on the peptide substrate (Huang et al., 2004; Ratia et al., 2006; Li et al., 2010; Zhang et al., 2020). The deprotonation of Cys145 facilitates its nucleophilic attack on the carbonyl carbon of glutamine in the polyprotein backbone to form a tetrahedral thiohemiketal intermediate, which also results in the formation of an oxyanion (Anand et al., 2002; Chuck et al., 2010; Kumar et al., 2017). The resulting oxyanion is stabilized by His41 and a nearby hydrogen bond donating amide groups of Gys143 and Ser144. Upon the collapse of the thiohemiketal into a thioester, the peptide bond is cleaved and the C-terminal part of the polyprotein substrate is released. Finally, a water molecule facilitates the hydrolysis of the thioester linkage for the release of the remaining N-terminal portion of the polyprotein substrate. This mechanism is reminiscent of that of the Ser–His–Asp catalytic triad found in chymotrypsin and other members of the serine protease family, with serine deprotonating the nucleophilic histidine, and aspartate facilitating the hydrolysis of the thioester linkage (Anand et al., 2003; Simon and Goodman, 2010; Ye et al., 2016). The role of the catalytic dyad His41 and Cys145 has not been analyzed for their effect on the activity, stability, and oligomerization for SARS-CoV-2 3CLpro.

Across coronaviruses, the active site and substrate binding site of 3CLpro is highly conserved. It is usually composed of substrate-binding subsites (S1′, S1, S2, S3, and S4) that accommodate residues (P1′, P1, P2, P3, and P4) of the peptide substrate (Dai et al., 2020; Lee et al., 2020). In particular, the correct conformation of the S1 substrate-binding subsite is dependent on the interactions between the two protomers. Residues at the S1 subsite are very important for the screening and development of antiviral drug targets, since the S1 subsite binds glutamine of the polyprotein substrate that is conserved in all coronaviruses (Li et al., 2016).

Previously, we have expressed and thermodynamically characterized SARS-CoV-2 3CLpro (Ferreira and Rabeh, 2020). The protease is most stable at physiological pH and favors low salt concentrations. Here, to further characterize the kinetic mechanism of 3CLpro, we expressed and characterized enzyme variants with different amino acid substitutions at the catalytic dyad (His41 and Cys145). As proposed here, detailed analysis of the roles of the substituted residues revealed their importance for SARS-CoV-2 3CLpro: not just for the catalytic activity but also for the overall conformational fold. These findings ensure the importance of targeting the cysteine–histidine pair in the development of antivirals against SARS-CoV-2 and other coronaviruses.

## Materials and Methods

### Construction and Expression of Recombinant 3CLpro Variants

The expression and purification of recombinant 3CLpro was described previously (Ferreira and Rabeh, 2020). Briefly, the expression of pET28b(+) vector containing 3CLpro in *Escherichia coli* BL21-CodonPlus-RIL (Stratagene) was initiated by the addition of 10 ml of an overnight starting culture in LB broth to 1 L of terrific broth supplemented with kanamycin and chloramphenicol (50 μg/ml final concentration of both). The culture was incubated at 30°C until the culture density reached OD_600_ of 0.8. Protein production was then induced by the addition of 0.5 mM isopropyl-β-d-1-thiogalactopyranoside and the culture incubated at 15°C for 14–16 h. The cells were harvested by centrifugation and resuspended in 30 ml of lysis buffer (50 mM Tris, pH 7.5, 150 mM NaCl, 5 mM imidazole, 3 mM β-mercaptoethanol, and 0.1% protease inhibitor cocktail from Sigma-Aldrich: P8849). The cells were lyzed by sonication and the supernatant was loaded at 4°C on a ProBond Nickel-chelating resin (Life Technologies) previously equilibrated with binding buffer (20 mM Tris, pH 7.5, 150 mM NaCl, 5 mM imidazole, and 3 mM β-mercaptoethanol). The column was washed with the same buffer supplemented with 75 mM imidazole, and the recombinant 3CLpro was eluted in the buffer supplemented with 300 mM imidazole. Pooled protein fractions were loaded onto a HiLoad Superdex 200 size-exclusion column equilibrated with 20 mM Hepes, pH 7.5, 150 mM NaCl, and 0.5 mM Tris(2-carboxyethyl)phosphine (TCEP), using an AKTA pure core system (Cytiva Life Sciences/Biacore). Protein sample purity was analyzed by SDS–PAGE. The samples were then concentrated by Amicon concentrator with 10-kDa membrane cut-off (Amicon, Inc., Beverly, MA, United States), for protein concentration of approximately 180 μM, as determined by Bradford assay.

### Analytical Size-Exclusion Chromatography and Circular Dichroism Analysis

The oligomeric state of 3CLpro wild-type (WT) and its variants was analyzed by analytical size-exclusion chromatography. Protein samples (180 μM protein in 50 μL) were injected onto Superdex 200 increase 10/300 GL using an AKTA pure core system (Cytiva Life Sciences/Biacore). The column was pre-equilibrated with 20 mM Hepes (pH 7.5). The flow rate was set at 0.75 ml/min and the samples were analyzed at 25°C. Each variant was analyzed three times to confirm the reliability of the data; the absorbance signal at 280 nm was normalized for the different variants.

The structural fold and conformational identity of 3CLpro variants were confirmed using CD spectroscopy. CD scans of the 3CLpro variants were collected in 100 mM phosphate buffer (pH 7.5) in the 190–260 nm wavelength range, and at a scanning rate of 10 nm/s, using a Chirascan CD spectrometer (Applied Photophysics), calibrated with aqueous camphor-10-sulfonic acid. For CD scans, 30 μM 3CLpro samples were used, and the CD signal was measured using a 1-mm quartz cuvette and a bandwidth of 1 nm, at 25°C.

### Kinetic Rate and Enzyme Titration Analysis of 3CLpro Variants

Proteolytic activity of 3CLpro was measured by monitoring the liberation of the fluorescent EDNAS group upon hydrolysis of the peptide substrate [DABCYL-KTSAVLQ↓SGFRKM-E(EDANS)-NH2, the hydrolysis site is indicated by ↓] in the presence of the donor and acceptor pair EDANS and DABCYL, respectively (Xue et al., 2007). The fluorogenic peptide was synthesized by GenScript (Piscataway, NJ, United States). The excitation and emission wavelengths were set at 360 and 500 nm, respectively, for the hydrolysis of the fluorescence resonance energy transfer (FRET) substrates, with the readings acquired using a Cytation 5 multi-mode microplate reader (Biotek Instruments, Winooski, VT, United States). The EDANS/DABCYL are donor–acceptor pair, where the excitation wavelength of 360 nm targets EDANS that emits light at 500 nm. In the undigested peptide where DABCYL is present close to EDANS, the emission of EDANS is quenched by DABCYL that absorbs light at 463 nm (Martins et al., 2019). The reaction was performed in a 96-well plate, with each well containing 50 μL of the peptide substrate. The plate was placed in a thermostatically controlled cell compartment at 30°C for 5 min; then, 50 μL of 3 μM 3CLpro variant was added. The reaction solution contained 20 mM Hepes (pH 7.0), 150 mM NaCl, 1 mM EDTA, 1 mM TCEP, and 20% (v/v) dimethyl sulfoxide (DMSO). The reaction rate calculated from the increase in the fluorescence signal was transformed into moles of hydrolyzed substrate per second using standard fluorescence curves of the peptide substrate. Enzyme titration measurements were done by varying the 3CLpro concentration (from 0.5 to 5.0 μM), at a fixed peptide substrate concentration (60 μM). The cleavage rate was obtained by fitting the initial data to a linear equation using SigmaPlot (Systat Software, San Jose, CA). Triplicate reactions were analyzed for each data point, and the values are displayed as the mean with standard deviation (SD).

### Differential Scanning Fluorimetry

Melting points of the 3CLpro variants were determined using DSF in the absence and presence of peptide substrate, with SYPRO Orange as a reporter dye with a final concentration of 5× was included in all melting assays. The reactions were performed in 96-well thin-walled PCR microplate (BioRad, Cat. No. 223 94444). The 3CLpro variants (25 μM) were prepared in 20 mM Hepes (pH 7.0), 150 mM NaCl, 1 mM EDTA, 1 mM TCEP, and 20% (v/v) DMSO, in the absence or presence of 50 μM peptide substrate. Thermal denaturation was initiated by increasing the temperature from 25 to 85°C at a heating rate of 1°C/min, using Mx3005P qPCR equipped with a Peltier-based thermal system (Agilent Technologies, La Jolla, CA). The thermal unfolding signal of proteins was assessed from the increase of the fluorescence intensity of 5× concentrated SYPRO Orange dye, with the excitation and emission wavelengths of 492 and 610 nm, respectively. The thermal unfolding data were then fitted to Boltzmann sigmoidal function to calculate the melting temperature (*T*_m_) value of the different 3CLpro variants in the midpoint of the thermal transition using the Excel add-on package XLft (IDBS limited, Bridgewater, NJ, United States), as described previously (Ferreira and Rabeh, 2020). In addition, the *T*_m_ of the WT and mutants of the 3CLpro was determined at different enzyme concentrations from 25 to 200 μM to evaluate the thermal stability for the monomeric and dimeric states of the protease.

### Differential Scanning Calorimetry

In addition to DSF, thermal stability of the different 3CLpro variants was assessed by DSC using Nano-DSC (TA Instruments, New Castle, DE, United States). The concentration of 3CLpro variants was maintained at 25 μM, in a buffer containing 20 mM Hepes (pH 7.0), 150 mM NaCl, 1 mM EDTA, 1 mM TCEP, and 20% (v/v) DMSO. The thermograms of 3CLpro variants were collected in the absence and presence of 50 μM peptide substrate; all samples were scanned from 15 to 75°C at the temperature ramp rate of 1°C/min. The buffer was used as a reference and the protein samples were degassed for 10 min prior to the start of each analysis run. The DSC scans were acquired by ramping up the temperature two times, to obtain two thermograms, where the second scan was used as the buffer background scan for each sample. The melting transitions of all 3CLpro samples were irreversible, as shown by a lack of signal from the second ramp up temperature scan. The DSC scans were normalized for protein concentration, and baseline-corrected by subtracting the corresponding buffer baseline. The data were then converted to plots of excess heat capacity (*C*_p_) as a function of temperature. 3CLpro *T*_m_ was determined from the temperature at the apex of the thermal transition, and calorimetric enthalpy (*ΔH*_cal_) of the transitions was estimated from the area under the thermal transition curve using NanoAnalyze Software v3.11.0 from TA instruments.

## Results

### Experimental Overview

**FIGURE 2:**
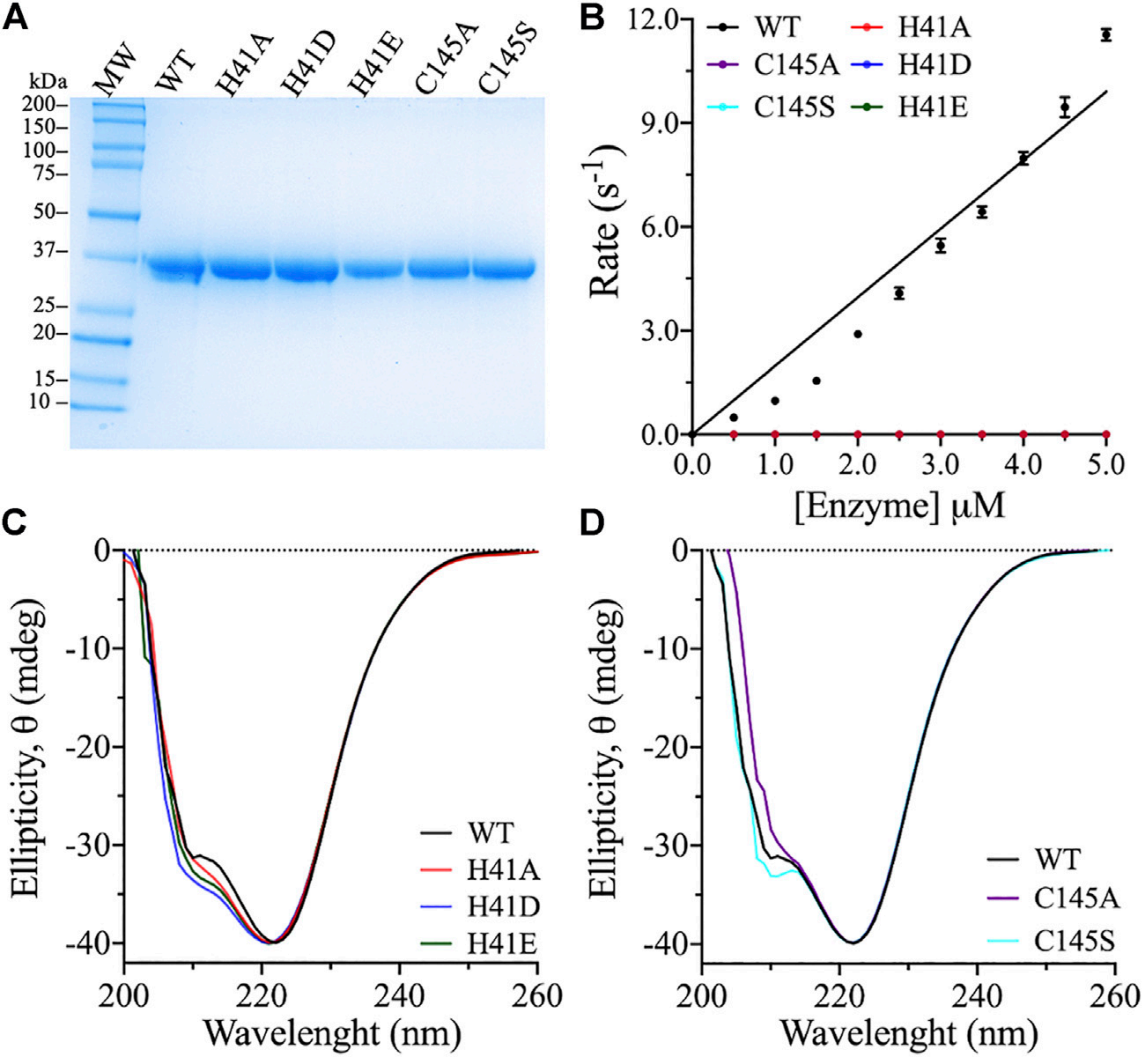
Purity, titration analysis, and structural fold conformation of 3CLpro variants. **(A)** The purity of 3CLpro WT and its variants was assessed by resolving around 25 μg protein on SDS–PAGE gel, with Coomassie Brilliant Blue staining for protein band visualization. The molecular mass marker and 3CLpro variants are as labeled with theoretical molecular weight of 38.5 kDa for the 3CLpro enzyme. The gel band densitometry was performed using ImageJ software. **(B)** Cleavage rates of the 3CLpro WT and variants at 30°C. The WT was the only active enzyme form. Data are presented as the mean ± SD, *n* = 3. **(C,D)** Far UV-CD spectra (260–200 nm) of the 3CLpro WT and variants. The CD scans were collected at 25°C in phosphate buffer (pH 7.5). Each scan is an average of five CD scans.

The 3CLpro catalytic dyad residues, His41 and Cys145, were substituted with different amino acids using site-directed mutagenesis using the 3CLpro gene previously cloned into pET28b(+) vector as a template (Ferreira and Rabeh, 2020). The different variants were expressed in *E. coli* and purified using immobilized nickel–nitrilotriacetic complex (Ni–NTA) affinity column, followed by size-exclusion chromatography. The purity of the protein samples was >90%, as estimated based on their resolution on an SDS–PAGE gel with molecular weight estimated at 38.5 kDa (Figure 2A). Structural integrity and the secondary structural fold of the 3CLpro variants were verified using far-UV CD analysis. The spectra of the catalytic dyad variants were similar to that of the WT enzyme (Figures 2C,D). The far-UV CD scans of the WT and the variants exhibited two ellipticity minima at 208 and 222 nm, similar to a chymotrypsin-like fold with a mixed α-helical and β-sheet structure (Chen et al., 2006; Ferreira and Rabeh, 2020). Small variations on the CD scans are observed at 208 nm with identical peaks at 222 nm, which indicate minimal effect on the overall secondary structure. The CD scans of the variants with different amino acid substitutions of the catalytic dyad residues confirmed high secondary structural identity with the WT, which confirmed a proper structural fold of the variant enzymes.

### Enzymatic Activity of Catalytic Dyad Variants

To determine the roles of the catalytic dyad residues in the catalytic activity of 3CLpro, alanines were introduced at His41 and Cys145 to eliminate the side chains of these residues. The cleavage rate of 3CLpro was measured using a highly sensitive FRET-based enzymatic assay (Anand et al., 2003; Kao et al., 2004; Kuo et al., 2004; Xue et al., 2007; Grum-Tokars et al., 2008; Hilgenfeld, 2014; Gioia et al., 2020; Jin et al., 2020; Zhang et al., 2020). The proteolytic rate of 3CLpro was monitored continuously by measuring the cleavage of a fluorescent peptide substrate. Because of its high sensitivity, the FRET-based enzymatic assay is one of the most common methods used for monitoring the proteolytic rate of 3CLpro. However, the peptide substrate contains many hydrophobic amino acid residues and hence, its solubility in water is low. To enhance the solubility and stability of the peptide substrate in the enzymatic assay, 20% (v/v) DMSO was included in all reactions, and the cleavage rate was monitored at 30°C. The proteolytic activity of the WT and the variants was measured at a fixed peptide substrate concentration (60 μM), while varying the enzyme concentration (0.5–5.0 μM). Increasing the enzyme concentration was important for ensuring the detection of the anticipated low enzyme activity of the variants. The WT enzyme exhibited increasing catalytic rate upon increasing the enzyme concentration with highest enzymatic rate of 12 s^−1^ was recorded at 5.0 μM enzyme concentration.

Alanine substitution at His41 and Cys145 inactivated the protease activity of 3CLpro, confirming their importance in catalysis of the peptide substrate cleavage (Figure 2B). Alternative substitutions at the catalytic dyad residues were also introduced, to test the retention of enzyme activity. His41 is important for the deprotonation of the catalytic Cys145 for its attack on the glutamine backbone at P1 site of the peptide substrate (Anand et al., 2002; Chuck et al., 2010; Kumar et al., 2017). Consequently, His41 was exchanged for aspartate and glutamate, as they were expected to function as general bases for the deprotonation of Cys145 in 3CLpro. However, the H41D and H41E variants did not exhibit any activity even at high enzyme concentration tested (5.0 μM; Figure 2B), and neither variant complemented the role of His41 in the catalytic mechanism. Further, Cys145 was substituted with serine, as in the catalytic triad of serine proteases in which serine acts as a nucleophile during catalysis (Hedstrom, 2002). However, serine substitution of Cys145 did not allow for the catalytic activity of 3CLpro (Figure 2B). In summary, all amino acid substitutions tested inactivated SARS-CoV-2 3CLpro.

### Effect of Catalytic Dyad Substitutions on the Oligomeric State of 3CLpro

**FIGURE 3:**
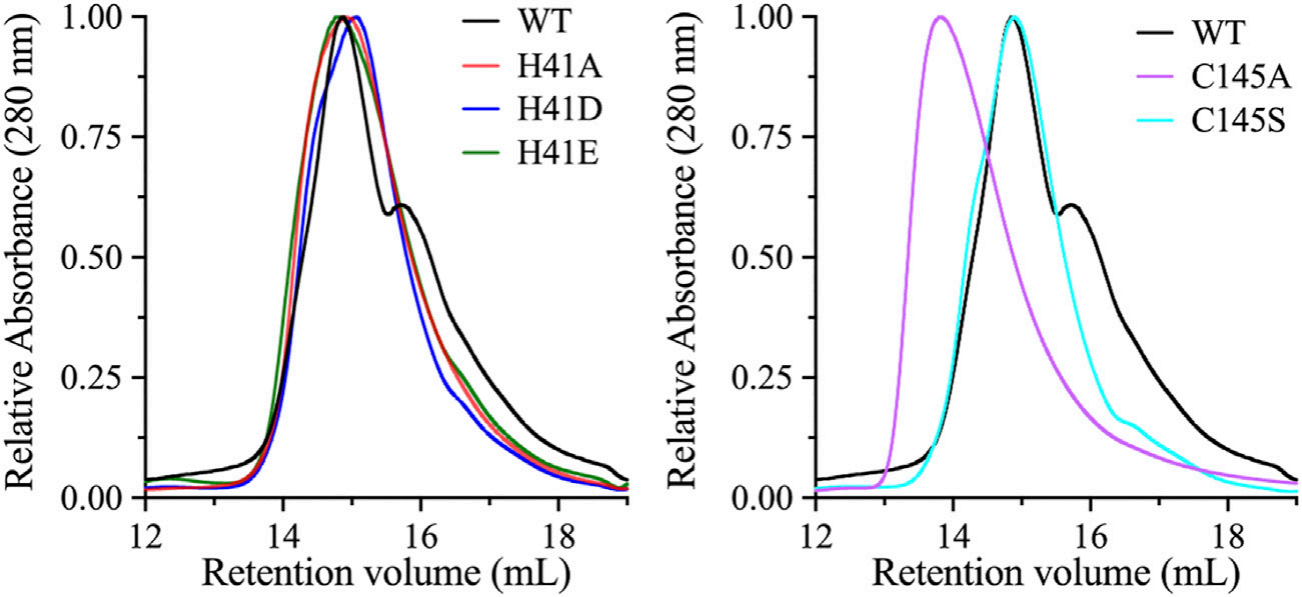
Oligomeric state analysis of 3CLpro variants. Size-exclusion chromatography profiles of the WT and its variants. Highly concentrated protein samples (180 μM) were injected onto the size-exclusion column (Superdex 200 increase 10/300 GL). The WT exhibited an early large peak corresponding to the dimeric state, with a late smaller peak corresponding to the monomeric state. The chromatographic peaks of His41 variants were different from those of the WT enzyme, with a single dominant peak of dimeric-state protein. Similarly, the chromatographic peak of the C145A variant exhibited a single peak that aligned with the dimeric peak of the WT enzyme. However, the single chromatographic peak of the C145S variant was shifted to an earlier retention volume, which represents a higher oligomeric state than the dimeric state of the WT enzyme.

The dimeric form of SARS-CoV 3CLpro is important for its catalytic activity, and the dimer interface interactions stabilize the appropriate substrate binding conformation (Hsu et al., 2005; Barrila et al., 2006; Chen et al., 2008; Barrila et al., 2010; Li et al., 2016). To investigate if the loss of catalytic activity of the active site variants is a result of the change in the dimeric state of the enzyme, molecular sizes of the variants were compared with that of the WT enzyme by Superdex 200 gel filtration chromatography. Initially, the WT enzyme did not exhibit a dimer-like chromatographic behavior and only after increasing the concentration of the injected protein to a relatively high value of 180 μM, a dimer-like peak appeared on the gel filtration chromatogram (Figure 3). Even though, high enzymatic rate was recorded at 5.0 μM, which indicated the presence of a dimeric state of the enzyme. In the gel filtration analysis, high protein concentration was used in an attempt to shift the majority of 3CLpro to the dimer state. This was expected, as it has been shown that increasing the concentration of 3CLpro shifts the equilibrium to the dimeric state (Zhang et al., 2020). The retention volume of the peaks from the gel filtration chromatogram indicated that WT 3CLpro formed both a monomer and a dimer, with the equilibrium shifted toward the dimeric state.

The enzyme variants were analyzed under same experimental conditions as the WT, including high protein concentration. The gel filtration profiles of the His41 variants revealed a dominant single peak corresponding to the dimeric state of 3CLpro, indicating that the introduction of alanine, aspartate, or glutamate at His41 stabilizes the dimeric state. The gel filtration profile of the C145S variant was similar to that of the His41 variants and the WT, with a single dimeric peak; however, the size of the single peak of the C145A variant was greater than that of the dimeric peak of the WT. Overall, with the exception of the C145A variant, the late peak corresponding to the monomeric state of 3CLpro disappeared in the gel filtration profiles of all His41 and Cys145 variants, which exhibited the same dimeric peak.

### Effect of Catalytic Dyad Substitutions on the Thermodynamic Stability of 3CLpro

**FIGURE 4:**
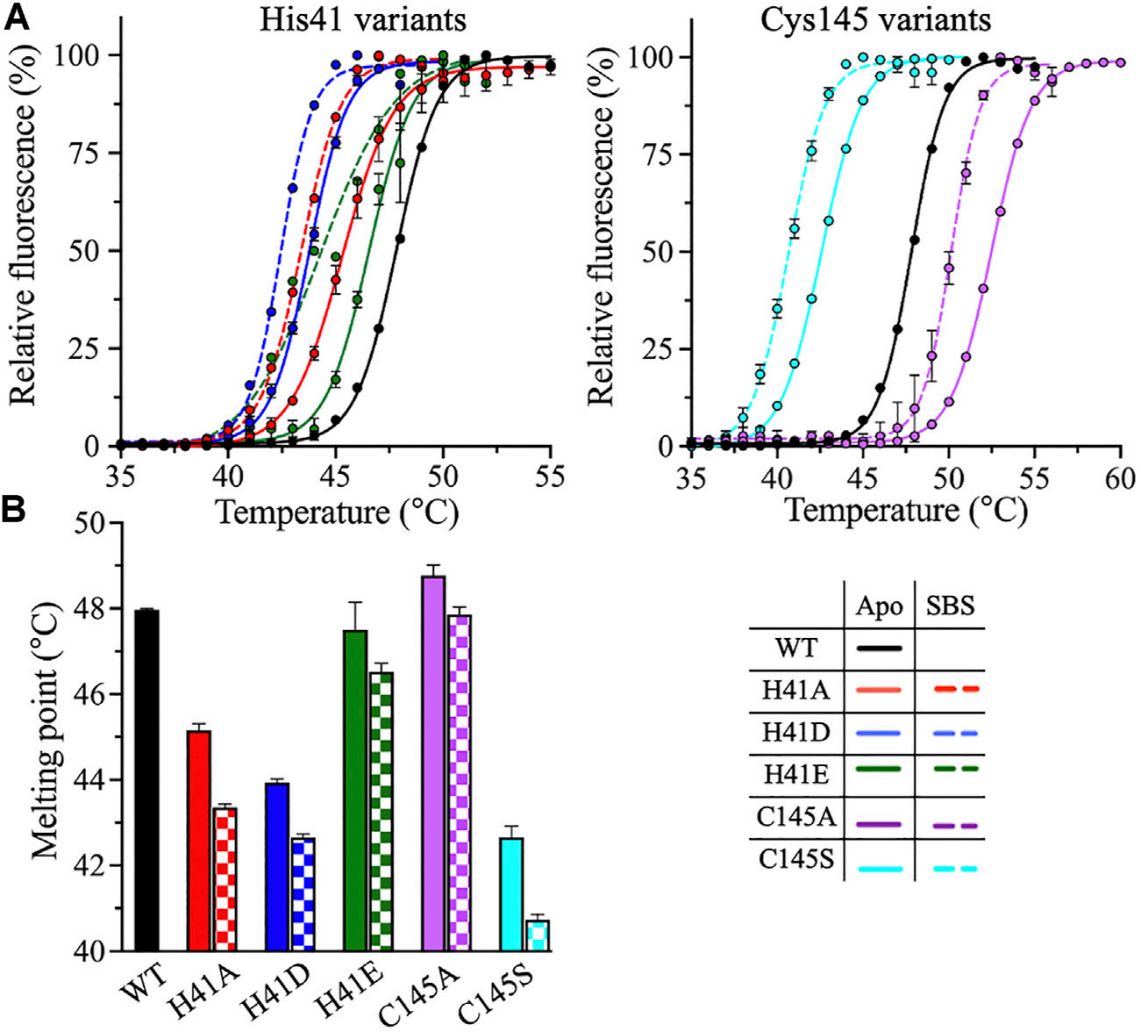
DSF measurements of 3CLpro variants. **(A)** DSF thermal scans of 3CLpro WT, and His41 and Cys145 variants acquired at pH 7.0 and 20% (v/v) DMSO, with SYPRO Orange as the reporter dye. The scans of the variants were acquired in the absence (solid line) or presence (dotted line) of 50 μM peptide substrate. The DSF scan of WT enzyme was not acquired in the presence of peptide substrate, as the protease is catalytically active. **(B)** Bar plot of *T*_m_ calculated from the midpoint of DSF thermal scans of 3CLpro in the absence (solid bars) or presence (checkered bars) of 50 μM peptide substrate. Colors are as in the bottom right panel. Data presented in **(B)** are shown as the mean + SD from, *n* = 3.

The thermodynamic stability of the catalytic dyad variants of 3CLpro was assessed using two thermoanalytical techniques: DSF and DSC. In DSF, the fluorescence signal of a protein increases upon protein unfolding in the presence of the reporter dye, SYPRO Orange. The DSF scans were obtained at pH 7.0 and 20% (v/v) DMSO, with a temperature ramping rate of 1°C/min. The *T*_m_ of 3CLpro was determined at the midpoint of the DSF thermal transition, where an increase in fluorescence is observed upon protein unfolding and the binding of the SYPRO Orange dye to the exposed hydrophobic core (Figure 4A). The introduction of alternative side chains at His41 decreased *T*_m_ from 48 ± 0.1°C, for the WT enzyme, to 45.2 ± 0.2 and 43.9 ± 0.1°C for the H41A and H41D variants, respectively (Figure 4B). *T*_m_ of the H41E variant was not affected. The only small increase in the 3CLpro stability was observed for the C145A variant (*T*_m_ of 48.8 ± 0.2°C). However, the largest drop, of 5.3°C, in *T*_m_ was observed for the C145S variant (*T*_m_ of 42.7 ± 0.3°C).

**FIGURE 5:**
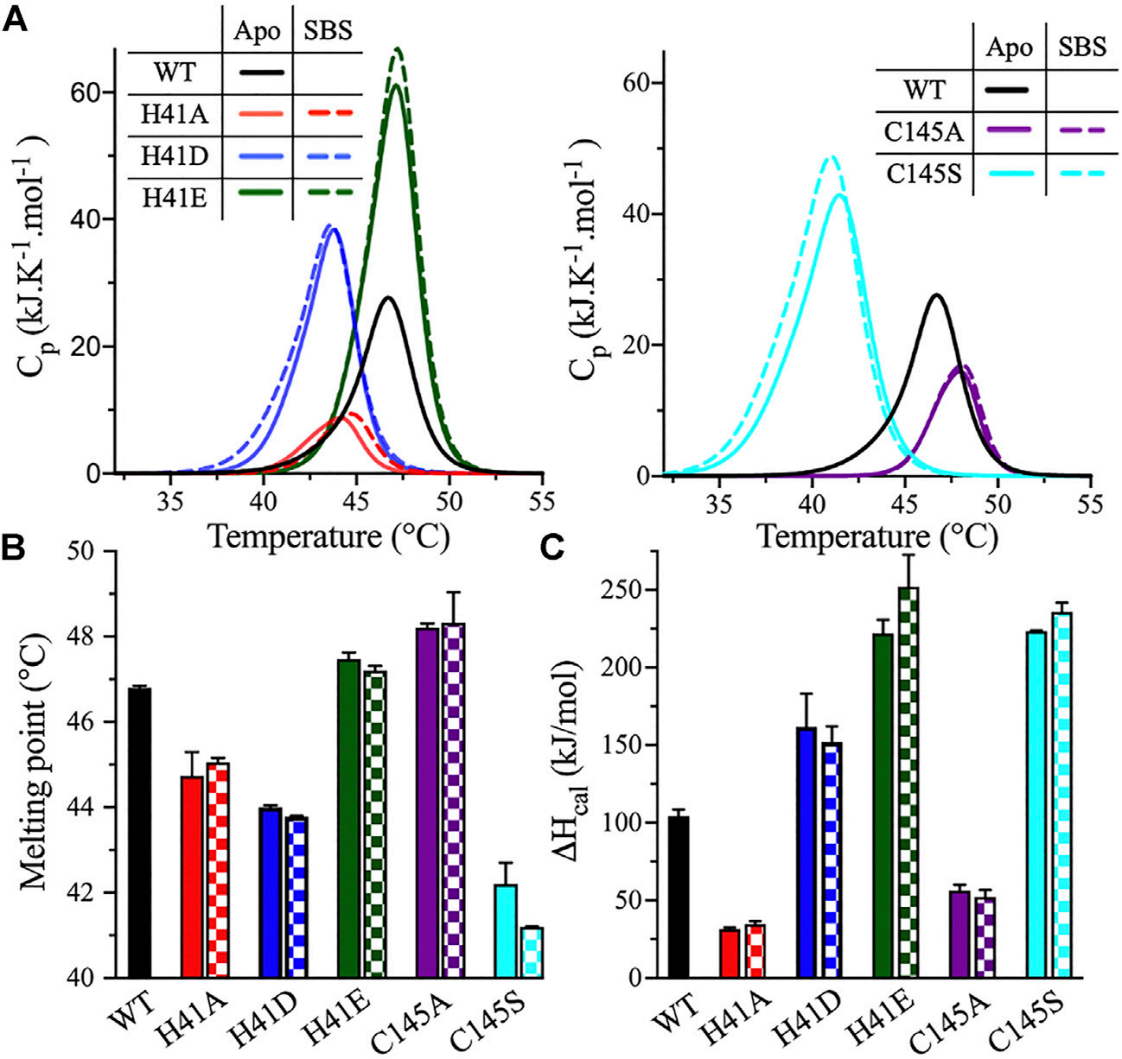
DSC measurements of 3CLpro variants. **(A)** DSC thermal scans of 3CLpro WT, and its His41 and Cys145 variants acquired at pH 7.0 and 20% (v/v) DMSO. The sample was heated at a rate of 1.0°C/min, and the scans of the variants were acquired in the absence (solid line) or presence (dotted line) of 50 μM peptide substrate. The DSC scan of WT enzyme was not acquired in the presence of peptide substrate, as the protease is catalytically active. **(B)** Bar plot of *T*_m_ calculated at the apex of the melting peak in the absence (solid bars) and presence (checkered bars) of 50 μM peptide substrate. Colors are as in panel A. **(C)** Bar plot of *ΔH*_cal_ calculated from the area under the thermographic peak in the absence (solid bars) and presence (checkered bars) of 50 μM peptide substrate. Colors are as in panel A. Data presented in **(B)** and **(C)** are shown as the mean + SD, *n* = 3.

The DSC analysis further confirmed the effect of the catalytic dyad substitutions observed with DSF. DSC thermograms of 3CLpro were acquired under the same conditions as the DSF experiments. The thermograms exhibited a single transition, and the *T*_m_ values were calculated at the apex of the melting peaks (Figure 5A). *T*_m_ decreased from 46.8 ± 0.1°C (WT) to 44.7 ± 0.6, 44.0 ± 0.1, and 42.2 ± 0.5°C, for the H41A, H41D, and C145S variants, respectively (Figure 5B). *T*_m_ of the H41E variant was unchanged relative to the WT and increased to 48.2 ± 0.1°C for the C145A variant, in close agreement with what was observed using DSF. Even though the shapes of the DSC scans of the variants were similar, the height and position of the peaks shifted in comparison with those of the WT enzyme. For the H41A variant, the thermographic peak shifted to a lower *T*_m_ value and the calorimetric enthalpy (*ΔH*_cal_) of unfolding decreased 3-fold, from 104 ± 4 kJ/mol for the WT enzyme to 32 ± 1 kJ/mol for the H41A variant (Figure 5C). By contrast, *ΔH*_cal_ for the H41D and H41E variants increased to 162 ± 21 and 222 ± 9 kJ/mol, respectively.

Cys145 substitutions resulted in an altered height and position of the DSC profile compared with the WT enzyme, but did not change its overall shape. The *ΔH*_cal_ values decreased 2-fold for the C145A variant, to 56 ± 4 kJ/mol; however, *ΔH*_cal_ increased 2-fold for the C145S variant, to 223 ± 1 kJ/mol (Figure 5C). The highest increase in the *ΔH*_cal_ value was observed for the H41E and C145S variants even though *T*_m_ of the former was not changed relative to the WT. The C145S variant had the most interesting DSC profile, with the highest drop in the *T*_m_ value (4.6°C) and the highest increase in the *ΔH*_cal_ value (119 ± 1 kJ/mol) among all variants tested (Figures 5B,C).

To evaluate the effect of peptide substrate binding on the stability of 3CLpro, the DSF and DSC scans of enzyme variants were acquired in the presence of 50 μM peptide substrate. The thermodynamic parameters of the substrate-bound states (SBS) of 3CLpro in the presence of the peptide substrate were compared to those of the apo state (i.e., in the absence of the peptide substrate). Based on the DSF analysis, the addition of the peptide substrate decreased *T*_m_ of all His41 and Cys145 variants, with an average drop from 1.0 to 2.0°C compared to the apo state (Figure 4B). However, the *T*_m_ values calculated from the DSC analysis revealed no major differences between the apo and SBS states of the variants (Figure 5B). In addition, the shape, position, and height of the thermographic peaks in the DSC scans of the enzyme variants in the absence and presence of the peptide substrate were similar (Figure 5A). Consequently, the *ΔH*_cal_ values of the variants did not change upon the addition of peptide substrate (Figure 5C). Of note, in the DSF analysis, the SYPRO Orange reporter dye, included to monitor the protein unfolding, may also interact with the peptide substrate because of the peptide’s hydrophobicity. As a result, the *T*_m_ values of 3CLpro variants in the SBS were less stable when measured by DSF; however, the values did not change in the DSC analysis. The binding interaction of the SYPRO Orange reporter dye to the peptide substrate may affected its ability to bind and stabilize the 3CLpro.

### Thermodynamic Stabilities of the Monomeric and Dimeric States of 3CLpro

**FIGURE 6:**
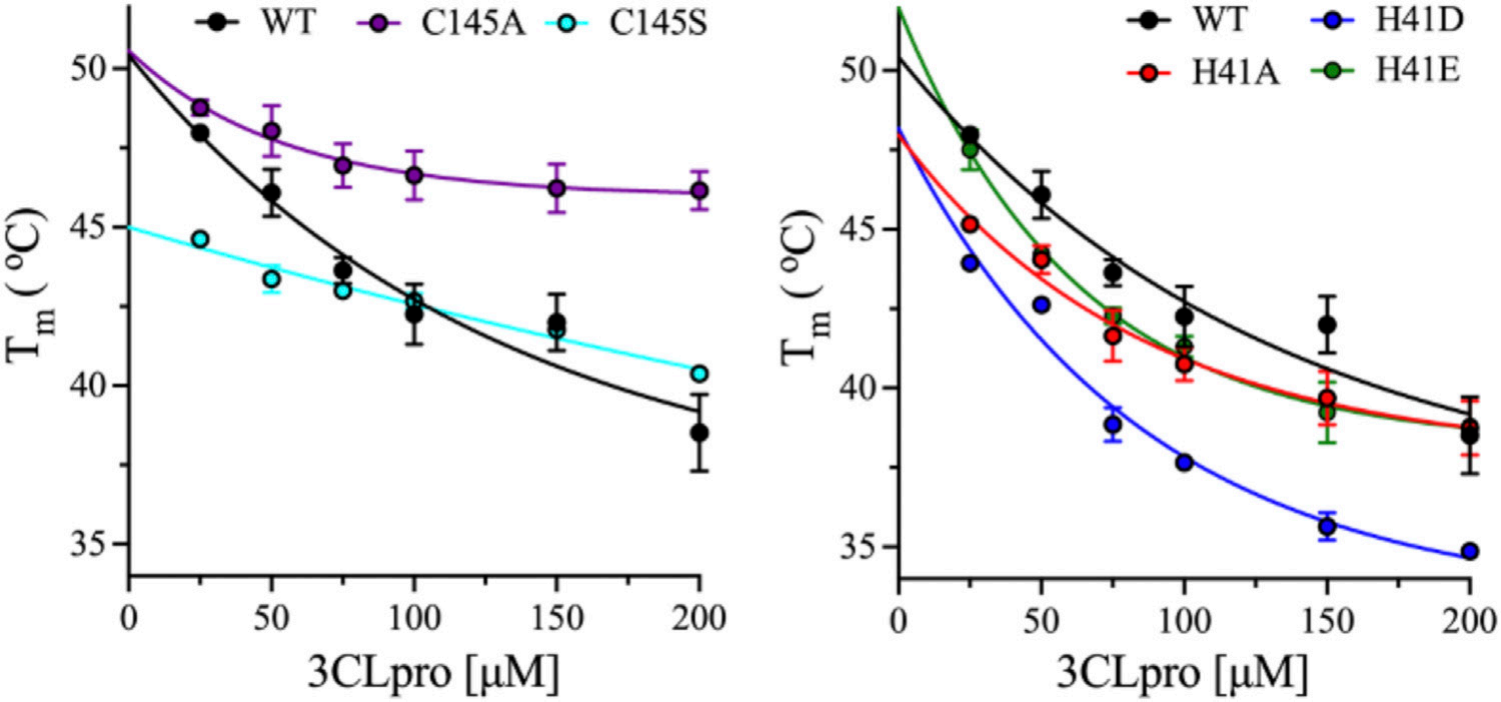
Effect of enzyme concentration on thermal stability of 3CLpro variants. Plots of *T*_m_ calculated from the DSF thermal scans of 3CLpro at different enzyme concentrations from 25 to 200 μM. Data are presented as the mean + SD from, *n* = 4.

To evaluate the thermal stability of the monomeric and dimeric states of 3CLpro variants, DSF was used to determine the *T*_m_ at different enzyme concentrations from 25 to 200 μM. At low and high enzyme concentrations, the equilibrium between the different oligomers of 3CLpro is shifted to monomeric and dimeric states, respectively. The dimeric state was detected at high protein concentration of 180 μM as discussed above. The *T*_m_ was determined from the thermal unfolding transition of 3CLpro in the presence of the reporter dye, SYPRO Orange at pH 7.0 and 20% (v/v) DMSO. The *T*_m_ for the WT enzyme decreased from 48 ± 0.1 to 38.5 ± 1.2°C upon increasing the protein concentration from 25 to 25 μM (Figure 6). The thermal stability of the WT decreased by 9.5°C upon shifting the oligomeric state from monomer to dimer for the 3CLpro. Similarly, the thermal ability decreased for variants at His41 with a decrease of 6.4°C was observed with H41A mutant and ∼9.0°C for H41D and H41E mutants (Figure 6). Increasing the enzyme concentration decreased the thermal stability of His41 mutants similar to the WT enzyme. However, smaller decrease in the *T*_m_ of Cys145 mutants was observed upon increasing the enzyme concentration with a drop of 2.6 and 4.3°C in the *T*_m_ of C145A and C145S mutants, respectively. Overall, the thermal stability of the dimeric state was lower than the monomers of 3CLpro for the WT and mutant enzymes tested here.

## Discussion

The 2019 outbreak of COVID-19 caused by SARS-CoV-2 has become a major health challenge worldwide. The development of antiviral agents against SARS-CoV-2 is essential for the treatment of COVID-19 and future outbreaks caused by other coronaviruses. The proteases of coronaviruses are vital targets for antiviral development, as they play crucial roles in the production and maturation of new virus particles. Hence, detailed understanding of the proteases’ reaction mechanisms will facilitate the screening of effective inhibitors against SARS-CoV-2 proteases to inhibit viral spread. The catalytic dyad His41 and Cys145 is important for the proteolytic activity of SARS-CoV-2 3CLpro. In the current study, these residues were substituted with different amino acids to alter the catalytic activity and stability of 3CLpro. To evaluate their role in catalysis, alanine was introduced at either position to eliminate the side chains of His41 and Cys145. The catalytic activity of 3CLpro was abolished in the H41A or C145A variants although CD analysis revealed that the abolished catalytic activity of these variants was not caused by changes in their secondary structure. Furthermore, the thermodynamic stability and *T*_m_ value of the H41A variant decreased; however, it did change in the C145A variant. On the other hand, *ΔH*_cal_ was significantly reduced by more than 2-fold in both these variants. The observed decrease of the *ΔH*_cal_ value can result from a partial exposure of the enzyme’s hydrophobic core in these variants (Arakawa et al., 2007) as the decrease in *ΔH*_cal_ is usually associated with an increase in protein hydrophobicity (Privalov and Khechinashvili, 1974; Arntfield and Murray, 1981; Molina Ortiz and Añón, 2001; Nawaz et al., 2018). Further, the oligomeric state of the H41A and C145S variants was the same as that of the WT enzyme, with a pronounced dimeric form. The C145A variant was the only variant whose oligomeric state was higher than that of the WT enzyme.

The thermodynamic stability of the different oligomeric states of WT and mutants of 3CLpro was investigated at different enzyme concentrations, where increasing the enzyme concentration would shift the equilibrium toward the dimeric state of 3CLpro. Surprisingly, for the WT and all mutants tested here, the dimer has lower thermodynamic stability than the monomeric form of 3CLpro as was observed from the DSF analysis. The drop in the *T*_m_ value of the His41 mutant was similar to the WT enzyme; however, the Cys145 mutants had lower drop in their *T*_m_ values. The lowest drop in the *T*_m_ value was observed with the C145A mutant which is also the only mutant that possess an oligomeric state higher than the dimeric form of the WT enzyme.

Other substitutions were introduced here to retain some of the activity of the WT enzyme, were also tested. Since His41 functions as a general base, it was substituted with aspartate and glutamate to complement its function in catalysis for the deprotonation of Cys145. Similar to the alanine substitution, the H41D and H41E variants did not exhibit any activity even at high enzyme concentration. The stability of 3CLpro was not affected by the H41E substitution and a considerable increase in the *ΔH*_cal_ value was noted for this variant. This indicates that polar bonding interactions contribute the most to the stability of the H41E variant. For the H41D variant, 3CLpro stability was reduced, with an increase in the *ΔH*_cal_ value. For both variants, the oligomeric equilibrium shifted in the direction of the dimeric state observed for the WT enzyme. Hence, the lack of catalytic activity upon H41D or H41E substitution is not a result of a disturbed structural fold of the enzyme but, rather, it is related to overall change of the conformational fold of the enzyme. Changes in the conformational fold of an enzyme alter the active site environment, affecting the catalytic behavior of the enzyme. Even though both aspartate and glutamate can in theory function as a general base and promote the 3CLpro-catalyzed reaction, the H41D and H41E variants were catalytically inactive.

Finally, serine was introduced at Cys145 as yet another amino acid substitution, to partially retain the catalytic activity of the enzyme. Serine is expected to function similarly to cysteine, i.e., as a nucleophile, in the 3CLpro catalytic reaction. Similar to other substitutions of the cysteine–histidine dyad, the serine substitution variant was not active. The stability of 3CLpro C145S slightly increased, with a major increase in the *ΔH*_cal_ value, similar to the effect of H41E. The oligomeric state of the C145S enzyme was similar to that of other variants, in that it preferentially adopted the dimeric state.

In serine proteases, a catalytic triad consisting of Asp-His-Ser plays an essential role in the cleaving ability of the proteases. The hydrogen bonding interactions of the carboxyl group on aspartate with the histidine of the catalytic triad is important in making the histidine a better nucleophile. The interactions facilitated by the aspartate increase the electronegativity of histidine making it capable to deprotonate the serine for its nucleophilic attack on the carbonyl carbon of the peptide substrate. However, in cystine proteases as the case for 3CLpro, an aspartic residue is not present to bind His41 of the catalytic dyad to make it a strong nucleophile for the deprotonation of serine substitution at Cys145. The side chain of cysteine has a *pK*_a_ value of ∼8.3 that is much lower than that of serine with *pK*_a_ > 13. Therefore, the deprotonation of the hydroxymethyl side chain of serine requires a much stronger nucleophile compare to the thiol side chain of cysteine. As a result, the serine substitution at Cys145 for 3CLpro did not exhibit any catalytic activity as it won’t be able to be deprotonated by His145.

Overall, the catalytic dyad is vital for the catalytic activity of SARS-CoV-2 3CLpro, and none of the amino acid substitutions tested resulted in an active enzyme. Based on the CD spectroscopy analysis, the enzyme variants maintained the secondary structural fold of the WT. Hence, the loss of activity was related to the importance of the side chain of the catalytic dyad residues for catalysis, as well as the environment of the active site. Supplementing the active site with amino acids that in principle can function similarly to the cysteine–histidine pair, while altering the microenvironment of the active site affected the catalytic ability of 3CLpro. The use of the catalytic dyad His41 and Cys145 in the design of antivirals against 3CLpro will yield effective drugs against COVID-19 and possible future coronaviruses outbreaks. There is a great need to develop effective and safe therapeutics against coronaviruses for the fast content of the current COVID-19 and future pandemics.

## Data Availability

The raw data supporting the conclusions of this article will be made available by the authors, without undue reservation.
